# Adaptation to the Speed of Biological Motion in Autism

**DOI:** 10.1007/s10803-019-04241-4

**Published:** 2019-10-19

**Authors:** Themis Karaminis, Roberto Arrighi, Georgia Forth, David Burr, Elizabeth Pellicano

**Affiliations:** 1 Department of Psychology, Edge Hill University, St Helens Rd, Ormskirk, L39 4QP UK; 2 Department of Neuroscience, Psychology, Pharmacology and Child Health, University of Florence, Viale Pieraccini 6, 50139 Florence, Italy; 3 Department of Child and Adolescent Psychiatry, Institute of Psychiatry, Psychology and Neuroscience, King’s College London, De Crespigny Park, London, SE5 8AF UK; 4 Institute of Neuroscience, National Research Council (CNR), Via Giuseppe Moruzzi 1, 56125 Pisa, Italy; 5 Department of Educational Studies, Macquarie University, Building X5B, Wally’s Walk, Sydney, NSW 2109 Australia; 6 Centre for Research in Autism and Education, UCL, London, UK; 7 Institute of Neuroscience, National Research Council (CNR), Pisa, Italy

**Keywords:** Autism, Perception, Adaptation, Biological motion, Running speed

## Abstract

Autistic individuals often present atypicalities in adaptation—the continuous recalibration of perceptual systems driven by recent sensory experiences. Here, we examined such atypicalities in human biological motion. We used a dual-task paradigm, including a running-speed discrimination task (‘comparing the speed of two running silhouettes’) and a change-detection task (‘detecting fixation-point shrinkages’) assessing attention. We tested 19 school-age autistic and 19 age- and ability-matched typical participants, also recording eye-movements. The two groups presented comparable speed-discrimination abilities and, unexpectedly, comparable adaptation. Accuracy in the change-detection task and the scatter of eye-fixations around the fixation point were also similar across groups. Yet, the scatter of fixations reliably predicted the magnitude of adaptation, demonstrating the importance of controlling for attention in adaptation studies.

**Electronic supplementary material:**

The online version of this article (10.1007/s10803-019-04241-4) contains supplementary material, which is available to authorized users.

## Introduction

Perceptual adaptation refers to the continuous recalibration of the response properties of perceptual and sensory systems driven by recent sensory experiences (Clifford and Rhodes 2005). For example, a quiet and continuous pure tone will be perceived to decrease in loudness over time (adaptation to loudness; see Lawson et al. 2015), while prolonged exposure to a face identity will cause a bias to perceive subsequently presented faces as dissimilar to it (adaptation to face identity; see Pellicano et al. 2007). Such adaptation is a ubiquitous property of perception and is thought to offer many functional advantages (e.g., Kohn 2007), in particular with regards to the efficiency with which sensory systems distinguish relevant from irrelevant stimuli. Limitations in adaptation should imply increases in the transmission of redundant information and should render individuals less able to distinguish relevant from irrelevant stimuli (Barlow 1990; Clifford et al. 2007; Webster et al. 2005). Such limitations could therefore have profound effects on how individuals perceive and interpret incoming sensory information.

Adaptation is also pertinent to theoretical accounts of autistic perception aiming to account for a range of sensory atypicalities and symptoms in the condition (DSM-5; American Psychiatric Association 2013). Atypicalities in perceptual adaptation have been thought to reflect difficulties of autistic^1^ individuals in deriving or using prior knowledge representations accrued from recent sensory experiences (Pellicano and Burr 2012). Within the Bayesian inference, or predictive-coding theoretical frameworks, which, in broad terms, suggest that the brain continually exploits the statistics of the world to predict current sensory input using a hierarchical and bidirectional processing system which aims to minimise prediction error within a cascade of cortical processing (Clark 2013; Friston 2010), adaptation may relate to the atypical encoding of precision in the perceptual hierarchy in autism (Lawson et al. 2014) or the inability to process flexibly prediction errors (Van de Cruys et al. 2014).

Given the ubiquitous presence of adaptation in perception, an intriguing possibility is that autistic individuals’ atypicalities in adaptation are pervasive across perceptual domains. The presence of domain-general atypicalities in adaptation could account for sensory issues in autistic people (e.g., why they might find certain sounds particularly disturbing), as well as core social difficulties, on the basis of a common neural mechanism (Lawson et al. 2018).

With regard to social stimuli, attenuated adaptation in autism has been observed consistently within the face-processing domain, including, for example, for facial identity in autistic children (Ewing et al. 2013b; Pellicano et al. 2007) and relatives of autistic children (Fiorentini et al. 2012), for facial configuration (Ewing et al. 2013a, b) and eye-gaze direction in children (Pellicano et al. 2013) and adults (Lawson et al. 2018), and for emotional expressions in children (Rhodes et al. 2018) and adults (Rutherford et al. 2012). van Boxtel et al. (2016) also found that autistic children show reduced adaptation to action discrimination in biological motion (walking vs. running).

Turning to the processing of non-social stimuli, autistic children have been found to present attenuated adaptation to numerosity (Turi et al. 2015) and, in the auditory domain, autistic adults have been found to present attenuated adaptation to loudness (Lawson et al. 2015) and audiovisual integration (Turi et al. 2016). Three studies, however, have failed to find evidence of atypical adaptive-coding abilities, including Cook et al. (2014), who reported intact adaptation to facial expression and identity in autistic adults, Karaminis et al. (2015), who found that autistic and typical children did not differ in the degree of adaptation of perceptual causality, and Maule et al. (2018), who found that autistic and typical adults did not differ in the degree of adaptation to colour.

In this study, we contribute new evidence about the adaptive coding of the speed of biological motion in autistic children and adolescents. The examination of the adaptive coding of biological motion in autism is important for two reasons. First, the processing of biological motion is key for a wide range of social competencies, such as inferring other people’s emotions, mood, and intentions (e.g., Brooks et al. 2008). Previous research on the abilities of autistic individuals to process biological motion stimuli has produced mixed results. Autistic individuals have been found to present reduced sensitivity to biological motion and atypical brain activation patterns following the presentation of relevant biological stimuli in some studies (Annaz et al. 2012; Blake et al. 2003; Freitag et al. 2008; Klin and Jones 2008; Koldewyn et al. 2010; Nackaerts et al. 2012; Wang et al. 2015; see also Wang et al. 2018, for a recent behavioural genetics approach), but other studies have found no such difficulties (Cusack et al. 2015; Edey et al. 2019; Jones et al. 2011; Murphy et al. 2009; Saygin et al. 2010; van Boxtel et al. 2016). With regard to the adaptive coding of biological motion in autism, van Boxtel et al. (2016) found attenuated adaptation to action discrimination in autistic children while action discrimination (per se) was intact. There are (to our knowledge) no other studies examining the adaptive coding of biological motion in autism beyond action discrimination (van Boxtel et al. 2016).

Second, it is important to examine the adaptive coding of biological motion in autism to establish whether findings for attenuated adaptation in autism during the processing of social stimuli are specific to faces or extend to other, high-level social stimuli. This could be likely as biological motion is supported by high-level neuronal mechanisms within the superior temporal gyrus (STS) and the fusiform and the lingua gyri (Gobbini et al. 2007; Vaina et al. 2001), that is, brain areas that are also involved in the processing of faces (Grossman et al. 2000), as well as the extrastriate and fusiform body areas (EBA and FBA; Jastorff and Orban 2009).

In this study, we used a different paradigm for biological motion from that used in the study by van Boxtel et al. (2016). Our paradigm focuses on adaptive coding of the speed of running silhouettes presented with point light displays (PLDs). We employed child- and autism-friendly methodologies and we also aimed to account for participants’ attention to the stimuli. This was important as earlier studies have shown that attention modulates the size of adaptation (Kreutzer et al. 2015; Rhodes et al. 2011). Controlling for attention was achieved by employing a dual-task paradigm, in which the primary task measured the perception of biological motion and adaptive coding, while the secondary task motivated participants to attend to the middle of the screen and assessed their attention (see also Ewing et al. 2013b; Karaminis et al. 2015; Lawson et al. 2018; Rhodes et al. 2018). We also collected eye-movement data to quantify participants’ looking preferences during the task.

## Method

### Participants

Participants demographics are shown in Table 1.

**Table 1:** Descriptive statistics for developmental variables for autistic and typical participants

Measures	Autistic participants	Typical participants	Statistical comparison
N	19	19	
Gender (n females:n males)	6:13	11:8	X^2^(2, N = 38) = 1.72, p = 0.18
Age (years)			
Mean (SD)	14.15 (2.84)	13.93 (3.80)	t(36) = 0.23, p = 0.87
Range	8.68–19.37	7.40–18.75	
Verbal IQ^a^			
Mean (SD)	104.68 (14.21)	105.47 (10.91)	t(36) = 0.19, p = 0.85
Range	70–126	83–130	
Performance IQ^a^			
Mean (SD)	103.21 (18.94)	103.21 (18.95)	t(36) = 0.20, p = 0.85
Range	75–132	76–139	
Full-Scale IQ^a^			
Mean (SD)	104.32 (16.57)	105.58 (12.65)	t(33.66) = 0.26, p = 0.79
Range	80–132	77–138	
ADOS-2 calibrated severity score^b^			
Mean (SD)	(N = 16) 4.75 (1.48)	n/a	n/a
Range	3–7		
SCQ score			
Mean (SD)	N = 17 21.24 (8.41)	N = 15 2.87 (3.35)	t(21.44) = 8.29, p < .001
Range	5–37	0–12	

*SCQ* Social Communication Questionnaire (score out of 40; Rutter et al. 2003)

^a^Verbal, Performance and Full-Scale IQ were measured using the Wechsler Abbreviated Scales of Intelligence-2nd edition (WASI-II; 2011)

^b^ADOS-2 calibrated severity scores obtained from Autism Diagnostic Observation Schedule-2 (Lord et al. 2012), scores range from 1 to 10, higher scores reflect greater autism severity

#### Autistic Participants

Nineteen autistic participants (6 girls) aged between 8.8 and 19.5 years (M = 14.15; SD = 2.84) were recruited via schools in London and community contacts. All autistic participants had an independent clinical diagnosis of an autism spectrum disorder and met the criteria for autism on the Autism Diagnostic Observation Schedule-2 (ADOS-2) (Lord et al. 2012; cut-off score = 7) or the Social Communication Questionnaire-Lifetime (SCQ; Rutter et al. 2003; cut-off score = 15) (see Corsello et al. 2007). All autistic participants were considered to be cognitively able, achieving scores ≥ 70 in the Wechsler Abbreviated Scales of Intelligence-2nd edition (WASI-II; 2011).

#### Typical Participants

Nineteen typically developing participants (10 girls), recruited from local London schools, were selected from a pool of 63 participants to match the group of autistic participants for chronological age, t(36) = 0.23, p = 0.87, gender, X^2^(2, N = 38) = 1.72, p = 0.18, as well as for performance IQ, t(36) = 0.20, p = 0.85; verbal IQ, t(36) = 0.19, p = 0.85; and full-scale IQ, t(33.66) = 0.26, p = 0.79, as measured by the Wechsler Abbreviated Scales of Intelligence-2nd edition (WASI-II; 2011). Parents of typical participants also completed the SCQ (N = 11). SCQ scores of typical participants ranged between 0 and 12 (M = 2.64, SD = 3.50), below the cut-off point for autism (score of 15; Rutter et al. 2003).

#### Exclusions

Seven additional participants (3 autistic, 4 typical) were tested but excluded because of poorly-fitting psychometric curves, as judged by 2 observers who were blind to any demographic details of the participants (exclusion criterion #1). One additional typical child was excluded due to an IQ score lower than the threshold of 70 in the WASI-II (Wechsler 2011) (exclusion criterion #2). Five additional autistic and two additional typical participants were excluded due to poor performance on the attentional task (exclusion criterion #3, see “Measurements and Analysis” section). Finally, one additional autistic boy was excluded because he did not fixate centre-screen during the experimental task (exclusion criterion #4, see “Measurements and Analysis” section).

#### General Procedure and Ethics

The study was conducted in accordance to the principles laid down in the Declaration of Helsinki. The UCL Institute of Education Research Ethics Committee approved all procedures. Parents of all participants gave their informed written consent prior to their child’s participation in the study and participants gave their verbal assent. Participants were tested individually in a quiet room at the Institute of Education. The WASI-II was administered on the same day, before or after the session. The ADOS-2 was administered either on the same day or on a separate occasion.

### Stimuli and Apparatus

**Fig. 1:**
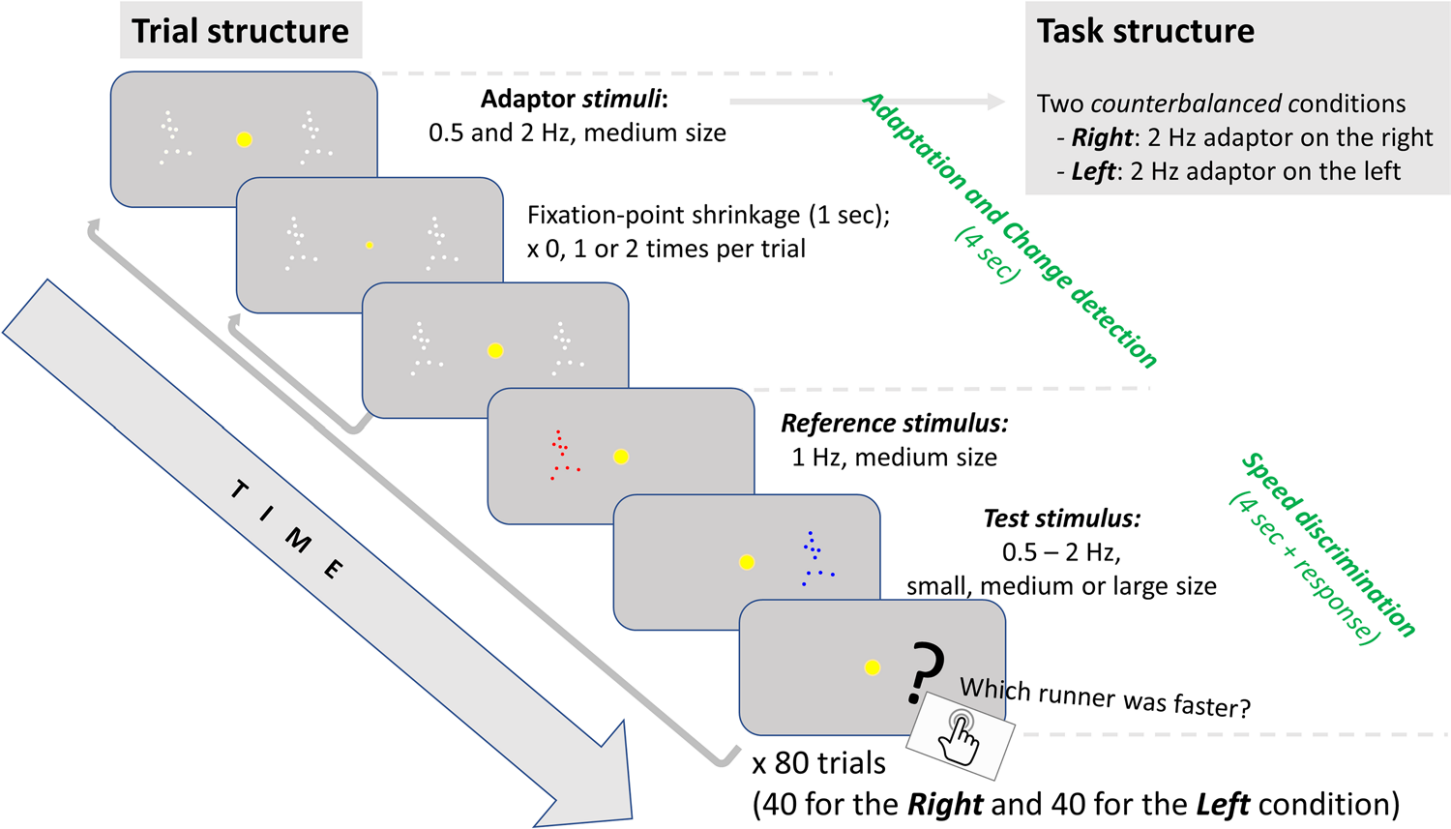
Trial structure and task design

Adaptor and test stimuli (see Fig. 1; see also Arrighi et al. 2010) were PLDs comprising 10 dots of diameter 0.75° of visual angle and simulating running human figures. An original version of PLDs stimulus representing the running human silhouette was downloaded from an online database (http://astro.temple.edu/~tshipley/ptltarchive.html; Shipley 2012). This movie displayed a complete running cycle (starting with the left foot on the floor and ending with the left foot landing again) in 20 frames. Using customised interpolation scripts, we created 6000 points within each running cycle. We defined running speed as the number of running cycles completed within a second (in Hz).

Adaptor and test stimuli appeared on the left- or the right-hand side of the screen (centred 10° from the centre of the screen). The adaptor stimuli were two PLDs which appeared in grey colour and in pairs, simultaneously on the right- and left-hand side of the screen for 4.0 s. The adaptor stimuli fitted a 10° height × 5° width frame (‘medium-sized’) and moved at a speed of either 0.5 Hz or 2 Hz.

The test stimuli were two PLDs, the Reference stimulus and the Test stimulus (see Fig. 1). The Reference stimulus appeared in red colour on the left-hand side of the screen for 2.0 s. It fitted a 10° × 5° frame (‘medium-sized’) and moved at a speed of 1 Hz. The Test stimulus appeared in blue colour on the right-hand side of the screen for 2.0 s. It appeared in three possible sizes: small (within a frame of 8° height × 4° width), medium (10° × 5° frame), or large (12° × 6° frame) and at different speeds at the range 0.5–2 Hz.

For the change-detection task, the main stimulus was a round dot subtending 1.0° in the centre of the screen, which occasionally shrank to a diameter of 0.75° twice during each adaptation period.

All stimuli were displayed on a 60 Hz TFT monitor measuring 50° × 28° when viewed at a distance of 57 cm, controlled by a Dell Desktop computer. The experiments were written in MatLab using routines of the Psychophysics Toolbox 3 (Brainard 1997; Pelli 1997; Kleiner et al. 2007). Eye-tracking data were collected using a Tobii-X300 eye tracker at 120 Hz and were processed with the Tobii Analytics Software Development Kit (SDK).

### Procedure

We measured perceptual adaptation to the speed of biological motion using a developmentally-sensitive computer game, which combined a speed-discrimination task, assessing adaptation to the speed of biological motion, and a change-detection task, motivating participants to attend to the centre of the screen. The general theme of the game was that participants were ‘Space Running Trainers’ aiming to form a winning team for the ‘Space Olympics’. To do so, participants should choose the fastest runners using a ‘specialised viewing machine’ (which provided the PLDs). The task structure and the trial structure are presented in Fig. 1.

#### Speed-Discrimination Task

The speed-discrimination task comprised two conditions, Right and Left (‘rounds’, counterbalanced across participants), each consisting of 40 trials presented in blocks (‘Levels’) of 13, 13, and 14 trials. Each trial included an adaptation phase, in which participants were exposed to adaptor stimuli, followed by a testing phase, in which participants judged the speed of test stimuli. The adaptation phase was differentiated in the Right and the Left condition so as to elicit adaptation aftereffects in two opposite directions (see also “Measurements and Analysis” section). The two conditions of the speed-discrimination task thus implemented a so-called ‘push–pull’ adaptation protocol.

In the adaptation phase, which lasted 4.0 s, participants watched the adaptor PLDs while they were encouraged to attend to the fixation point centre-screen (see also “Change-Detection Task” section). In the Right condition, the speed of the right adaptor PLD was 2 Hz, four times faster than the left adaptor (0.5 Hz). Conversely, in the Left condition, the right adaptor that ran at 0.5 Hz and the left at 2 Hz.

In the test phase, participants were presented with the two test PLDs, first the Reference stimulus on the left-hand-side of the screen and then the Test stimulus on the right-hand-side of the screen, for 2.0 s each. They were asked to indicate which runner they thought was the fastest by pressing a corresponding red or blue key on the keyboard. Responses were not registered until both runners had finished running.

The speed of the Reference PLD always was set at 1.0 Hz. The speed of the Test PLD was chosen using two QUEST functions (Watson and Pelli 1983), one starting at 0.5 Hz and ascending and one starting at 2.0 Hz and descending. The two QUESTs homed in on the point where the speed of the two test stimuli appeared equal; to ensure a good distribution of durations to estimate discrimination thresholds, a random jitter of SD = 0.1 log units was also added to the QUEST estimates (Watson and Pelli 1983).

The Test stimulus appeared in three possible sizes, small (8° × 4°), medium (10° × 5°), and large (12° × 6°). This manipulation ensured that our participants could not solve the discrimination task by relying on the local speed of the dots constituting the PLDs (see also van Boxtel and Lu 2013). For example, let’s assume that the two test stimuli (Reference and Test) moved at the same speed (say, a gait cycle per second) and that the Test stimulus was small. Because of this size difference, the distance covered by the individual dots of the Test stimulus (e.g., the feet) during a cycle gait would be shorter than the distance covered by the corresponding dots of the Reference stimulus. Based on this difference, if participants relied on a local-speed response strategy, they should present a bias to respond that the Reference stimulus would be faster. By contrast, if participants relied on a global response strategy, they should not present this bias.

#### Change-Detection Task

In the change-detection task, participants were asked to respond to changes of the fixation (‘viewing machine losing power’) point by pressing the spacebar (‘powering up the machine’). The fixation point returned to normal after a response. The change-detection task took place during the adaptation phase of the trials of the speed discrimination task. There were zero, one or two shrinkage events in each trial, each lasting 1 s.

#### Practice Trials and Motivation

Participants were given visual and verbal instructions for both tasks at the start of the game, including practice on pressing the spacebar when the dot in the centre of the screen shrank. They also completed eight practice trials, in which the speed of each of the running figures in the testing phase were very clearly different from each other (0.5 Hz vs. 1.5 Hz or 2.0 Hz). Practice trials were repeated if participants made more than three mistakes or if they responded that they needed more practice to proceed to the actual game. This happened only for two autistic participants and never more than once. Participants had the opportunity to take short breaks at the end of the testing blocks. They were regularly praised for their performance and, at the end of each round, they were shown a leaderboard. The experimenter encouraged them to attend to the centre of the screen throughout testing and monitored their attention.

### Measurements and Analysis

#### Speed-Discrimination Task

**Fig. 2:**
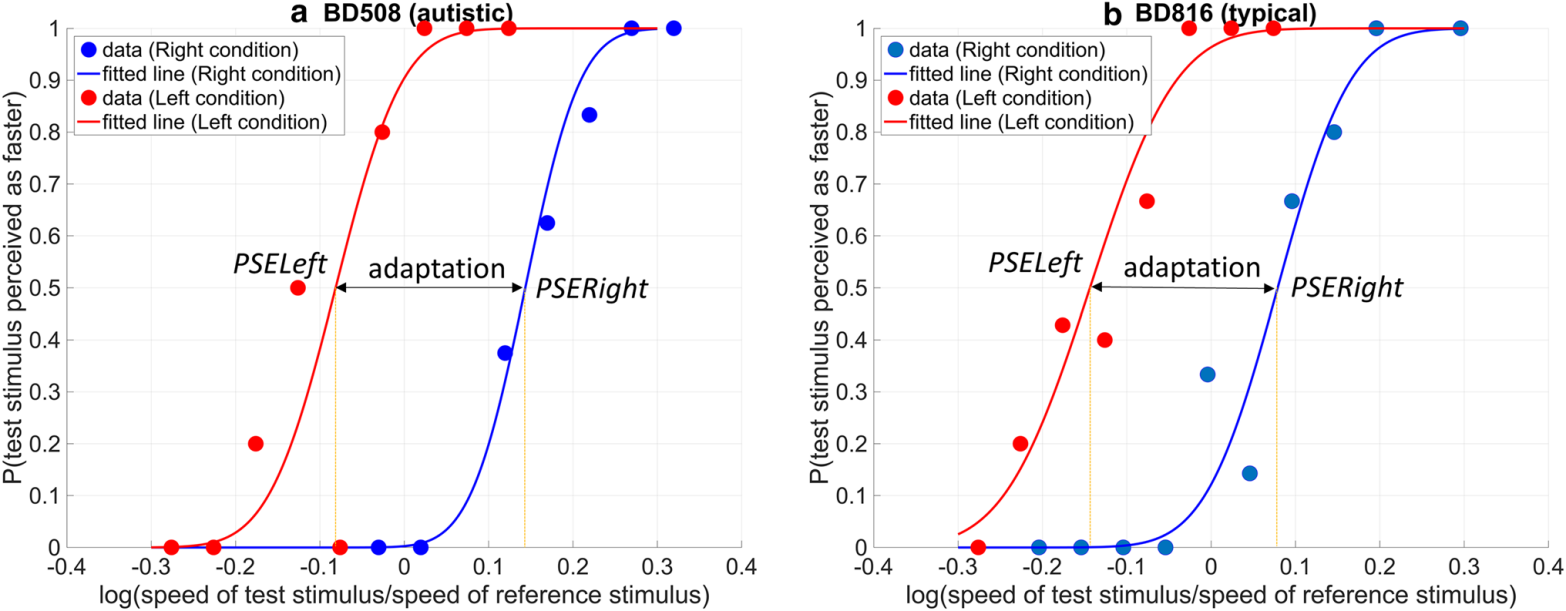
Sample data from an autistic and a typical participant and fitted psychometric curves. Adaptation is measured as the difference between the Points of Subjective Equality (PSE) in the Right and the Left condition

Figure 2 shows example data from two of our participants from the speed-discrimination task. We fitted individual data from participants with cumulative Gaussian functions using bootstrapping (Efron and Tibshirani 1993) with 10 repetitions and a ‘maximum likelihood’ fitting method (Watson 1981). First, two observers, blind to any demographic details, judged the quality of the fitted curves. Participants with poorly fitting curves were excluded from the analysis. From the fitted curves, and for each condition, we derived Weber Fractions [the standard deviations of the fitted Gaussians or just noticeable difference (JND) divided by the Points of Subjective Equality (PSE)] and the PSEs (the mean of the fitted Gaussians).

Weber Fractions provided an estimate of the precision with which participants judged the speed of the PLDs. We compared Weber Fractions using a repeated-measures ANOVA with Condition (‘Left’ vs. ‘Right’) as a between-participants factor and Group (‘Autistic’ vs. ‘Typical’).

The PSE corresponded to the value of the Test stimulus intensity (more precisely, the value of the log-transformed ratio *speed of Test stimulus*: *speed of Reference stimulus*) for which the judgements of participants in the speed-discrimination task were at chance levels, that is, participants responded that the Test PLD was faster than the Reference PLD with a probability of 0.5. For each participant, we derived *PSE_Right* and the *PSE_Left* using data from the Right and the Left condition, correspondingly. In our data, due to adaptation, *PSE_Right* tended to be higher than *PSE_Left.* This was as in the Left (Right) condition, the Test stimulus was presented after exposure to a slow (fast) adaptor and was thus perceived to be faster (slower), pushing (pulling) the psychometric curve to the left (right) (see Fig. 2). To estimate the magnitude of the adaptation effect we calculated the distance *PSE_Right *− *PSE_Left*. We compared the magnitude of adaptation in the two matched groups with an independent samples t-tests. We also performed a complementary Bayesian independent samples *t* test for this difference.

#### Change-Detection Task

For the change-detection task, we calculated mean accuracy (the proportion of detected shrinkages) in the change-detection task across both conditions. Participants with accuracy scores lower than 25% were excluded from the analysis. We also examined reaction times in the change-detection task (online measure).

#### Eye-Tracking Data

From the eye tracking data, we calculated the scatter of fixations around the centre of the screen (the standard deviation of average distance from the centre of the screen) during the adaptation and the testing phase. One autistic participant, with a scatter of fixation of 15.0° of the visual angle was excluded from the analysis. We also calculated correlations between the scatter of fixations and adaptation in the speed-discrimination task.

#### Correlational Analysis

In a secondary analysis, we examined correlations between adaptation to the speed of biological motion and precision in speed discrimination, as well as correlations between adaptation and demographic and eye-tracking variables.

## Results

### Similar Speed-Discrimination Precision and Similar Adaptation to the Speed of Biological Motion

**Fig. 3:**
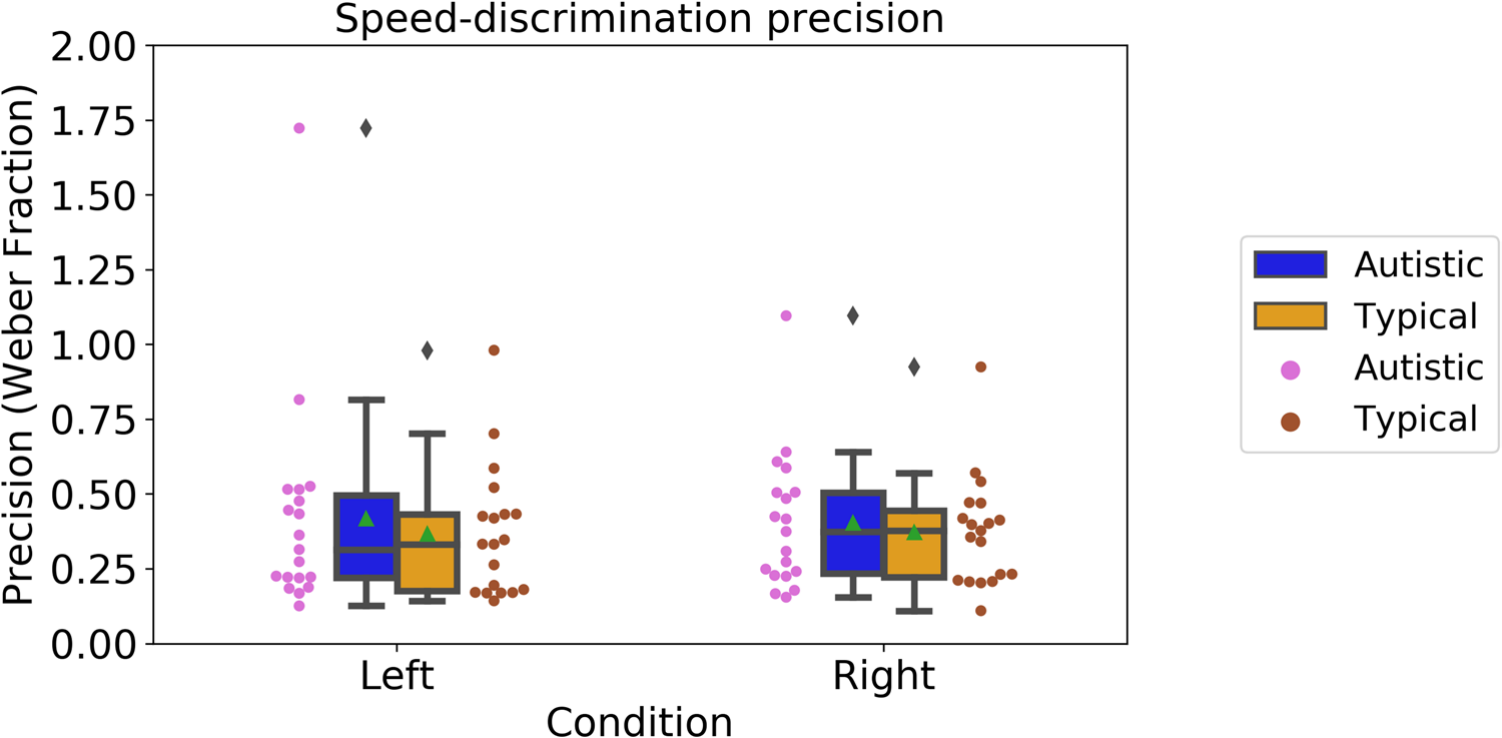
Speed-discrimination abilities of autistic and typical participants in the ‘Left’ and the ‘Right’ condition. Boxplots show group averages (green triangles) and medians (horizontal lines), dots show the performance of individual participants

First, we looked at precision in discriminating the speed of biological motion, expressed as Weber Fractions. Figure 3 shows Weber Fractions in the two conditions of the speed discrimination tasks (Left, autistic: M = 0.40, SD = 0.23; typical: M = 0.37, SD = 0.22; Right, autistic: M = 0.42, SD = 0.36; typical: M = 0.37, SD = 0.18). We conducted a mixed-design ANOVA with Group (‘Autistic’ vs. ‘Typical’) as a between-participants factor and Condition (‘Left’ vs. ‘Right’) as a within-participants factor. There were no significant effects of Group, F(1, 36) = 0.39, p = 0.54, *n*_*p*_^2^ = 0.01; Condition, F(1, 36) = 0.01, p = 0.93, *n*_*p*_^2^ < 0.01; and no significant interaction between the two factors, F(1, 36) = 0.04, p = 0.84, *n*_*p*_^2^ = 0.001. Our analysis therefore suggested that autistic and typical participants presented similar precision in speed-discrimination.

**Fig. 4:**
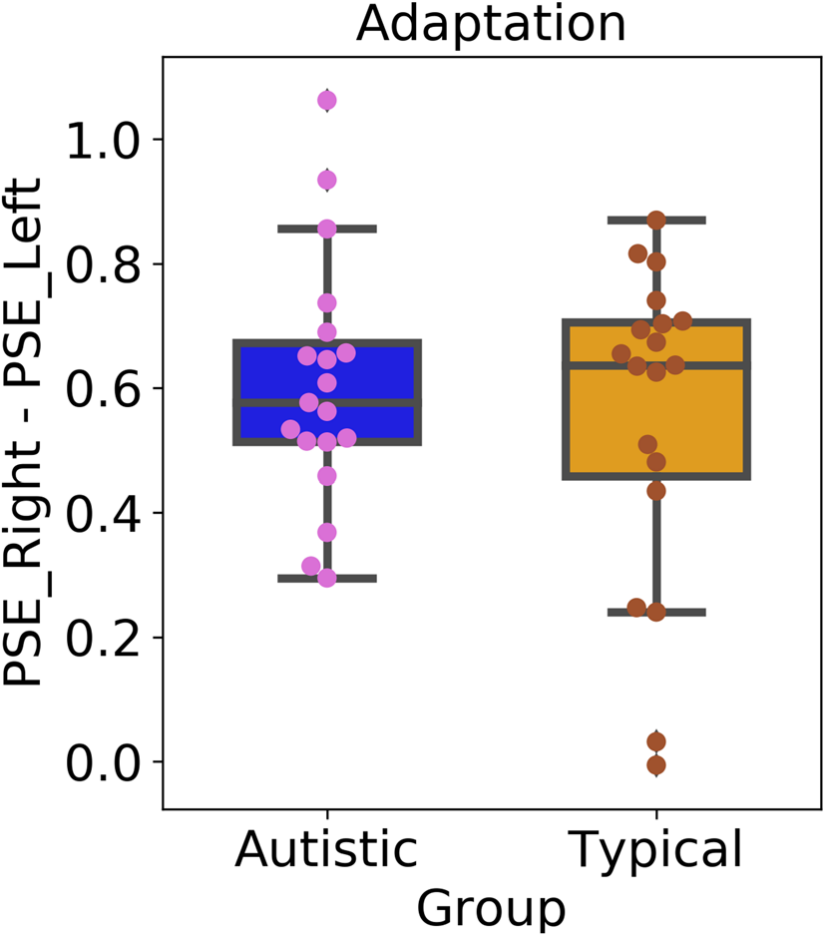
Adaptation to the speed of biological motion as measured by the difference between the Points of Subjective Equality (PSE) in the left and the right . Boxplots show group averages (green triangles) and medians (horizontal lines), dots show performance of individual participants

Next, we examined the magnitude of adaptation, shown in Fig. 4 (autistic participants: M = 0.60, SD = 0.20; typical participants: M = 0.55, SD = 0.26). The magnitude of the adaptation effect was significantly higher than 0 in both groups of participants, as revealed by one-sample t-test [autistic participants: t(18) = 13.36, p < 0.001; typical participants: t(18) = 10.77, p < 0.001]. Importantly, and contrary to our prediction, there were no differences in adaptation between autistic and typical participants, t(36) = 0.50, p = 0.48, *d* = 0.20.

We also performed a Bayesian independent samples t-test using JASP software (Version 0.8.0.0; JASP Team 2016) and estimated a Bayes factor using Bayesian information criteria (Wagenmakers 2007), which allowed for a comparison of the fit of our data under the null hypothesis that there are no differences between autistic and typical children in the magnitude of the adaptation to the speed of biological motion, and the alternative hypothesis that adaptation differs in the two groups of participants. The Bayes factor (null/alternative-estimated using a Cauchy distribution prior with a scaling factor of 1) was 3.38, suggesting that our results were 3.38 times more likely to occur under the null hypothesis than under the alternative hypothesis. Our data, therefore, provided substantial evidence (Wetzels et al. 2011) that autistic and typical participants adapted to the speed of biological motion to a comparable degree.

### Similar Performance in the Change-Detection Task

**Fig. 5:**
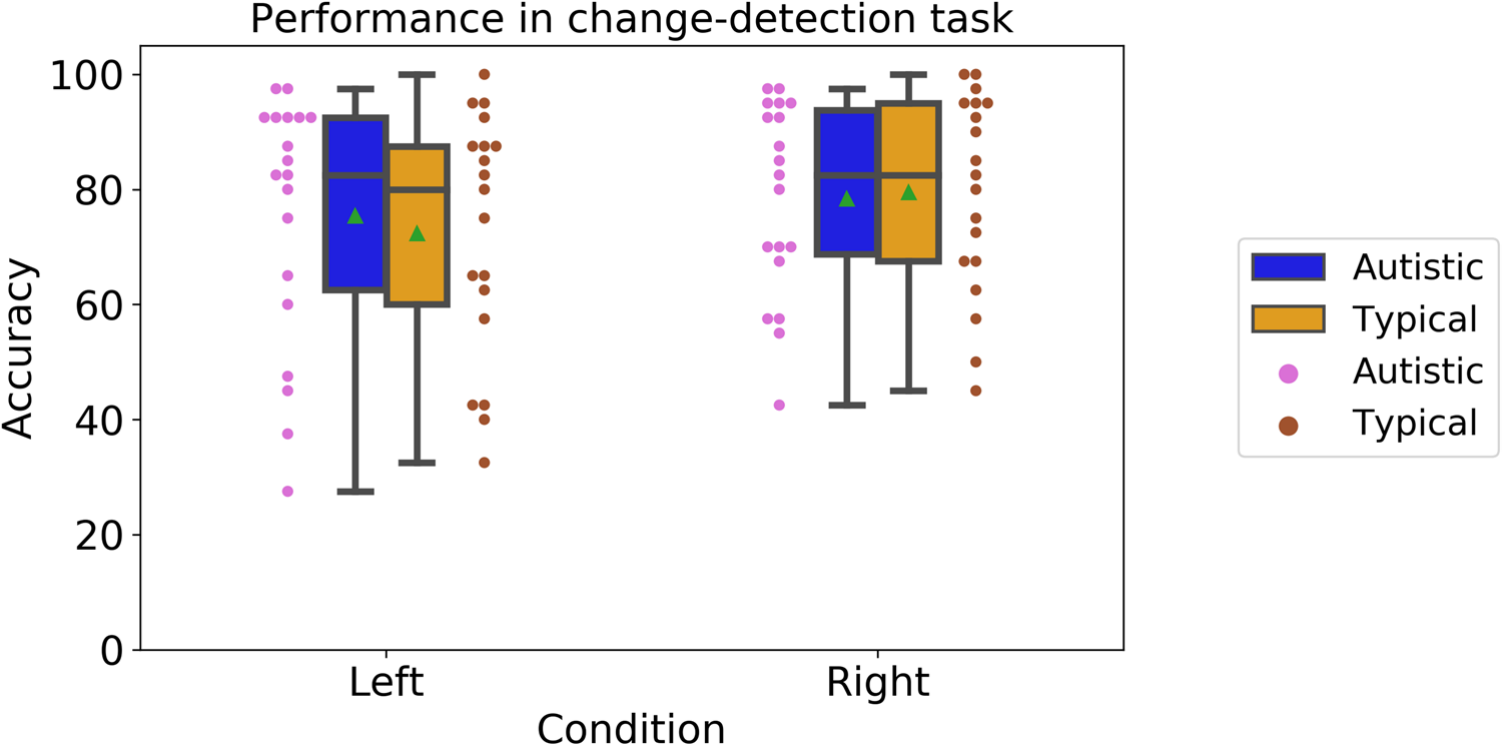
Accuracy in the change-detection task, in the two conditions. Boxplots show group averages (green triangles) and medians (horizontal lines), dots show performance of individual participants

Turning to the change-detection task, Fig. 5 shows accuracy rates in the two conditions of the task (Left, autistic: M = 0.78, SD = 0.17; typical: M = 0.79, SD = 0.17; Right, autistic: M = 0.75, SD = 0.22; typical: M = 0.72, SD = 0.21). A mixed-design ANOVA with Group (‘Autistic’ vs. ‘Typical’) as a between-participants factor and Condition (‘Left’ vs. ‘Right’) as a within-participants factor showed no effects of Group, F(1, 36) = 0.27, p = 0.87, *n*_*p*_^2^= 0.001, a significant effect of Condition, F(1, 36) = 6.16, p = 0.02, *n*_*p*_^2^= 0.15, and no significant interaction between Condition and Group, F(1, 36) = 0.99, p = 0.32, *n*_*p*_^2^= 0.00. Autistic and typical participants performed similarly on the secondary task.

### Similar Reaction Times in the Change-Detection Task

**Fig. 6:**
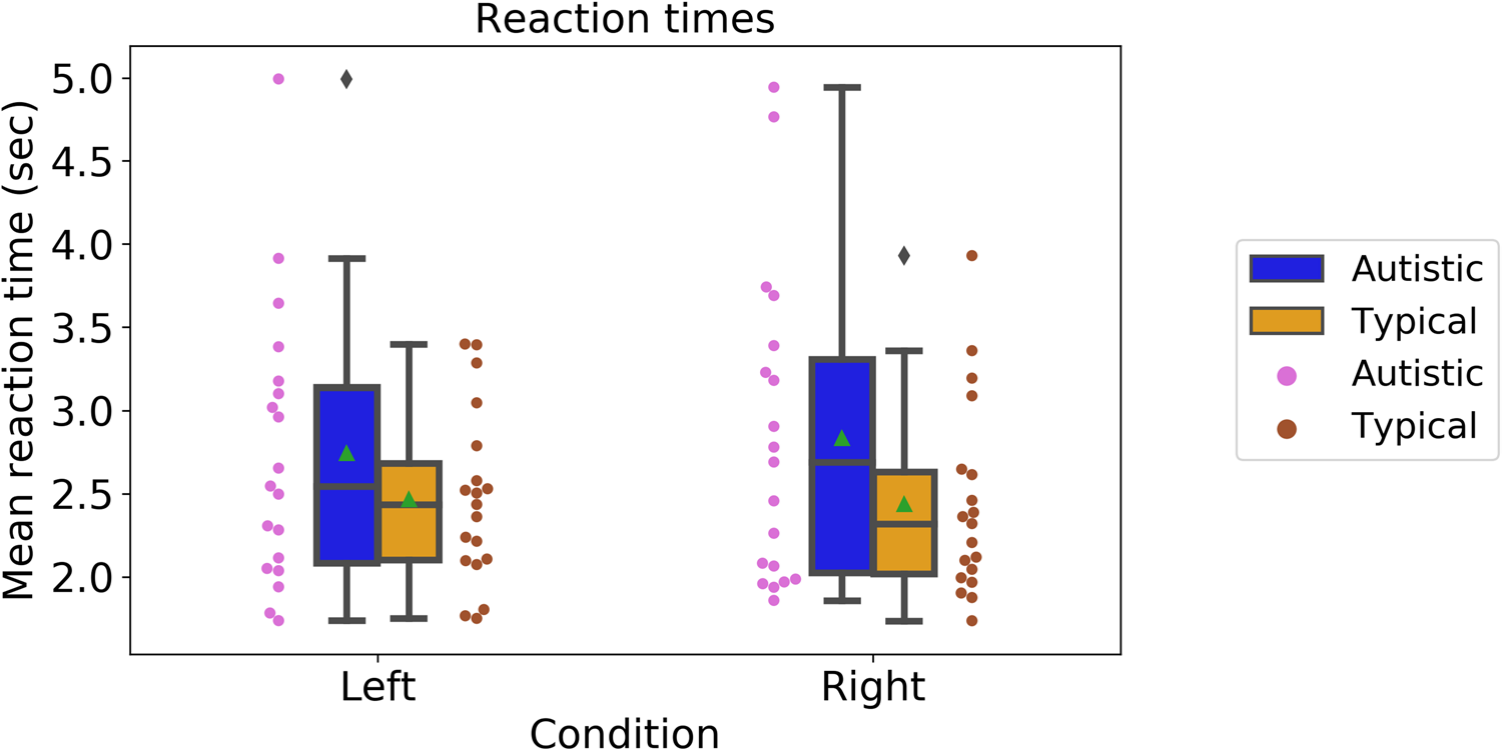
Reaction times in the speed-discrimination task. Boxplots show group averages (green triangles) and medians (horizontal lines), dots show the performance of individual participants

For the change-detection task, we examined mean reaction times, shown in Fig. 6 (Left, autistic: M = 2.84, SD = 0.94; typical: M = 2.74, SD = 0.83; Right, autistic: M = 2.74, SD = 0.83; typical: M = 2.47, SD = 0.52). A mixed-design ANOVA with Group (‘Autistic’ vs. ‘Typical’) as the between-participants factor and Condition (‘Left’ vs. ‘Right’) as the within-participants factor showed no significant effects of Group, F(1, 36) = 2.40, p = 0.13, *n*_*p*_^2^ = 0.06, or Condition, F(1, 36) = 0.12, p = 0.73, *n*_*p*_^2^ = 0.003, or condition × group interaction, F(1, 36) = 0.36, p = 0.54, *n*_*p*_^2^= 0.01. The results therefore suggested that autistic and typical participants did not differ in their reaction times.

### Similar Eye-Movement Data

**Fig. 7:**
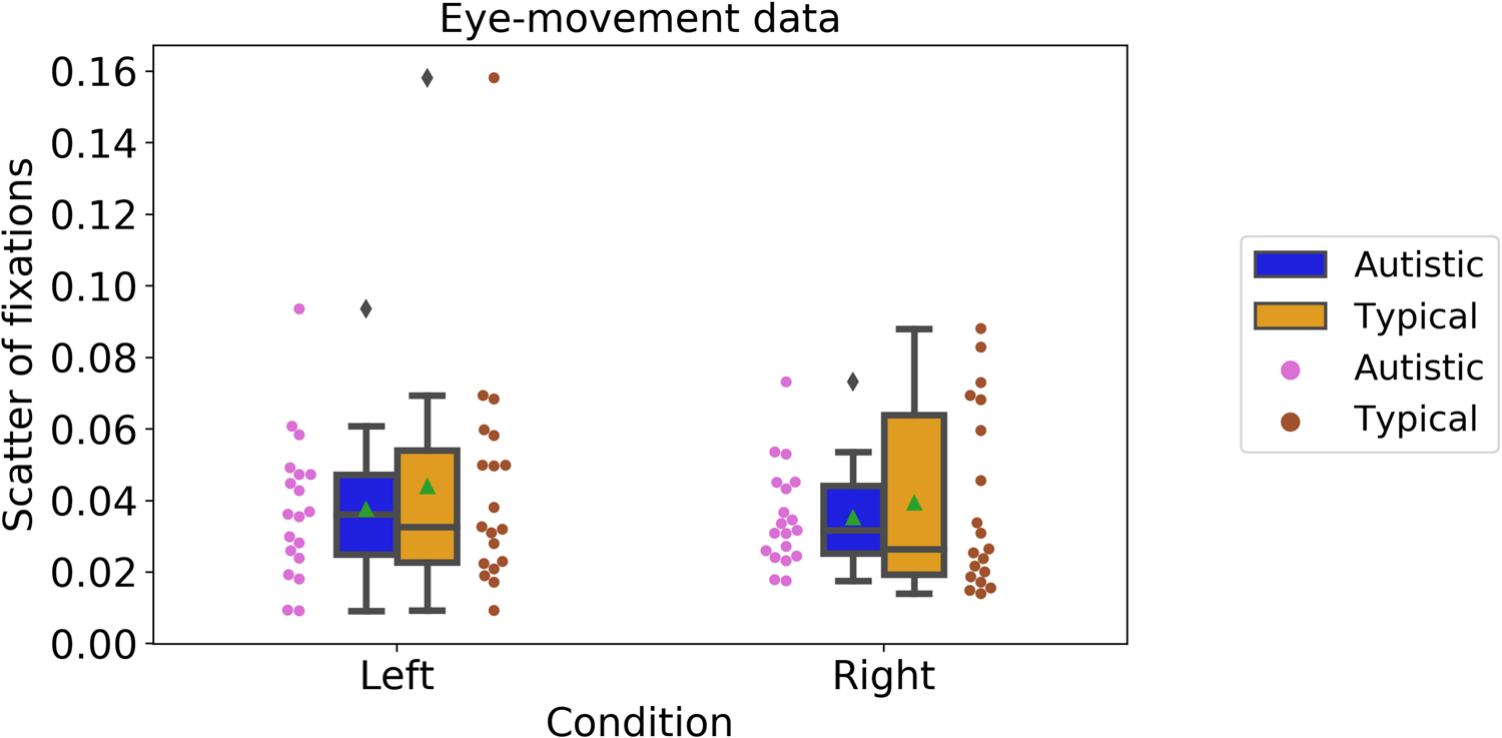
Scatter of fixations in the two conditions of the speed-discrimination task. Boxplots show group averages (green triangles) and medians (horizontal lines), dots show performance of individual participants

We also examined eye-tracking data to obtain an objective measure of the extent to which participants attended to the centre of the screen (as motivated by the change-detection task, as well as by the experimenter during the testing session). Figure 7 shows the scatter of fixations around centre-screen in the two conditions (in degrees of the visual angle) (Left, autistic: M = 0.035, SD = 0.014; typical: M = 0.039, SD = 0.025; Right, autistic: M = 0.038, SD = 0.020; typical: M = 0.044, SD = 0.032). Again, a mixed-design ANOVA with Group (‘Autistic’ vs. ‘Typical’) as a between-participants factor and Condition (‘Left’ vs. ‘Right’) as a within-participants factor and showed no significant effects of Group, F(1, 36) = 0.54, p = 0.47, *n*_*p*_^2^ = 0.02, Condition, F(1, 36) = 1.08, p = 0.31, *n*_*p*_^2^ = 0.03, and no significant interaction, F(1, 36) = 0.11, p = 0.74, *n*_*p*_^2^ = 0.00. Autistic and typical participants fixated to centre-screen to a comparable extent.

### Correlational Analysis

**Fig. 8:**
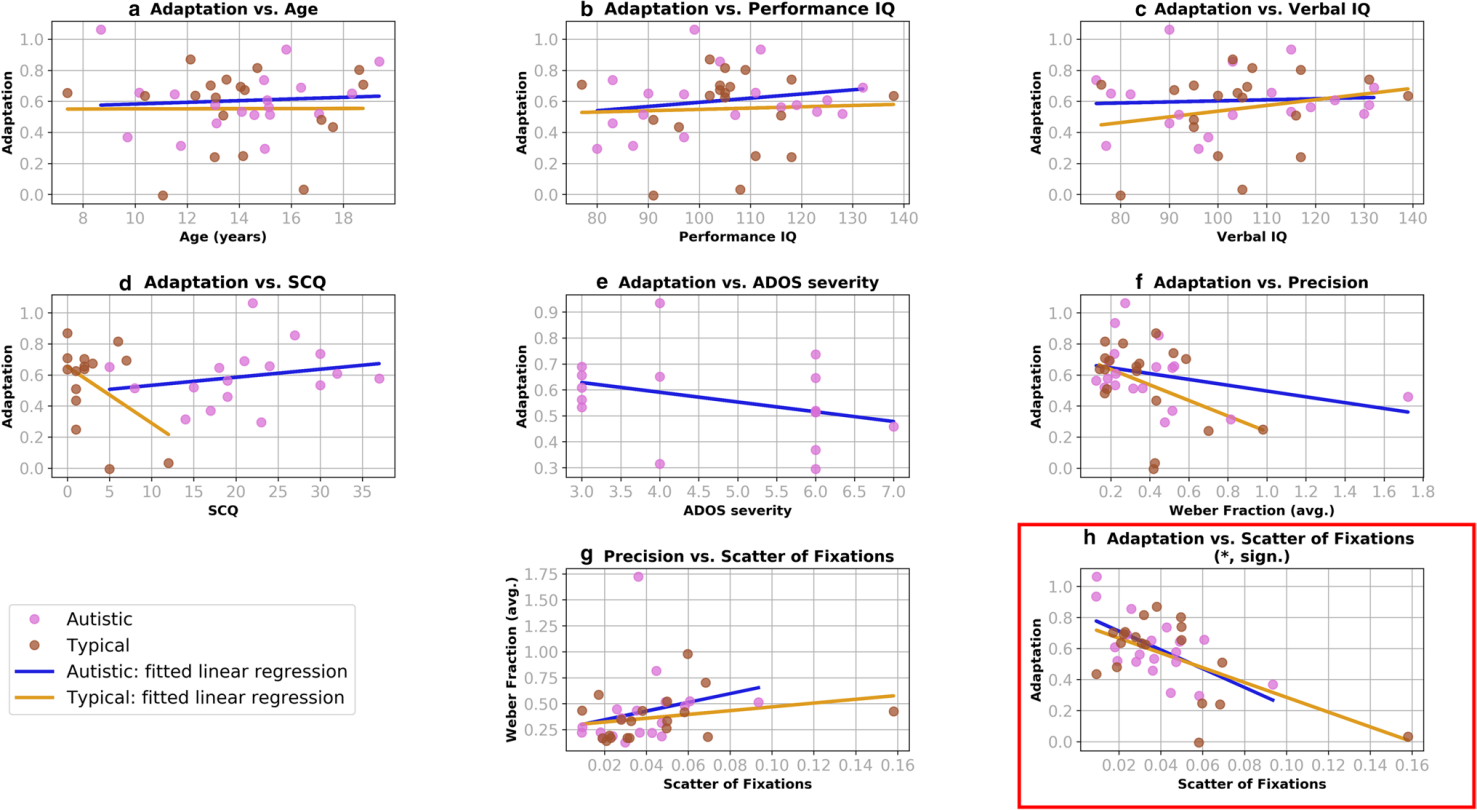
Results of the secondary correlational analysis of individual variability. Panels show correlations between the magnitude of adaptation and age (**a**), Performance IQ (**b**), Verbal IQ (**c**), scores on the SCQ (**d**), ADOS severity scores (**e**) and precision in the speed-discrimination task (**f**), as well as correlations between precision in the speed-discrimination task and the scatter of fixations (**g**) and correlations between adaptation and the scatter of fixations (**h**). The analysis suggested that in both groups of participants, the magnitude of adaptation was smaller for participants with more scattered fixations (panel **h**). Note that this relationship remained significant when the extreme value in the typical group was removed

In a secondary correlational analysis, we examined the relationship between adaptation to the speed of biological motion and precision in speed discrimination, as well as between and adaptation demographic and eye-tracking variables (Fig. 8). Correlations between adaptation to the speed of biological motion and precision were non-significant in either group of participants [autistic: r(19) = − 0.17, p = 0.55; typical: r(19) = − 0.36, p = 0.13]. Furthermore, in either group of participants, there were no significant correlations between adaptation and age [autistic: r (19) = 0.08, p = 0.78; typical: r(19) = 0.04, p = 0.99], and Performance-IQ [autistic: r (19) = 0.06, p = 0.79; typical: r(19) = 0.22, p = 0.36] and Verbal-IQ [autistic: r(19) = 0.36, p = 0.13; typical: r(19) = − 0.18, p = 0.46]. Within the group autistic participants, there were also no significant correlations between the magnitude of adaptation and autistic features, as indexed by ADOS-2 calibrated severity scores, r(16) = − 0.35, p = 0.18, or SCQ scores, r(17) = 0.23, p = 0.38. Correlations between the magnitude of adaptation and SCQ scores were also not significant when autistic and typical participants were considered as one group, r(28) = 0.08, p = 0.66.

Interestingly, there was a significant correlation between the magnitude of adaptation and the eye-tracking variable of the scatter of fixations in both autistic, r(19) = − 0.62, p = 0.005, and typical participants, r(19) = − 0.61, p = 0.01. As shown in Fig. 8h, the adaptation effect is less pronounced for participants who attended to a lesser extent to centre-screen. Note that correlations between the eye-movement measure and precision in speed-discrimination [autistic: r(19) = 0.15, p = 0.54; typical: r(19) = 0.34, p = 0.16] were non-significant.

## Discussion

In this study, we compared autistic and typical participants, of similar age and ability, on the adaptive coding of the speed of biological motion. We hypothesised that autistic individuals’ atypicalities in the adaptive coding of facial stimuli (Ewing et al. 2013a; Lawson et al. 2018; Pellicano et al. 2013; Rhodes et al. 2018; Rutherford et al. 2012) should generalise to non-facial social stimuli and predicted that autistic participants should show less adaptation to the speed of the PLDs of our task than the typical comparison participants. We found that both groups showed significant adaptation effects—but, contrary to our prediction, that the magnitude of adaptation was comparable in autistic and typical participants. This finding could not be attributed to group differences in attention or to looking differences, as both accuracy on the change-detection task and the scatter-of-fixations measure were similar across groups.

Furthermore, the lack of differences in adaptation between autistic and typical participants could not be due to differences in precision in speed discrimination. We found that the two groups were equally precise. This latter result is consistent with studies that do not find differences in the processing of biological motion in autism (Cusack et al. 2015; Jones et al. 2011; Murphy et al. 2009; Saygin et al. 2010; van Boxtel et al. 2016) rather than those that report reduced sensitivity and differences in the brain activation patterns to biological stimuli (Annaz et al. 2012; Blake et al. 2003; Freitag et al. 2008; Klin and Jones 2008; Koldewyn et al. 2010; Nackaerts et al. 2012).

Our results are also inconsistent with the study on adaptation to biological motion by van Boxtel et al. (2016), which examined a similar number of autistic and typical children. It is possible that this discrepancy is due to the focus on different aspects of biological motion (“running speed” vs. discrimination of type of movement in van Boxtel et al. 2016). It is difficult to understand the origin of these discrepancies without further investigation of performance in different types of biological motion within the same individual. It would be interesting to replicate our and van Boxtel et al.’s methods, also considering other biological motion characteristics such as gender, which is more explicitly social and to which adaptation has previously been shown in non-autistic adults (Jordan et al. 2006; Troje et al. 2006).

Another factor that could be considered in future studies is the likely correspondence between the kinematics of the test stimuli and the kinematics of participants. One study has reported that autistic adults present atypical kinematics and that the degree of such atypicalities predicts performance in a biological motion perception task (Cook et al. 2013). It is possible that the perceptual similarity or dissimilarity between the kinematics of stimuli and participants could also affect the adaptive coding of biological motion.

One important methodological feature of our study is that it carefully examined differences in attention. This was achieved by including the secondary change-detection task and using eye-tracking. By contrast, in van Boxtel et al. (2016), where autistic children were found to present attenuated adaptation, “the experimenter monitored fixation throughout the experiment, providing reminders as deemed necessary” (p. 4). Arguably, the use of a change-detection task is a more robust method for directing participants’ attention to the fixation point. Interestingly, the post hoc analysis of the eye-tracking data showed that the more participants attended to the fixation point, the larger the magnitude of adaptation. Therefore, even though autistic participants did not differ on average from typical participants on the degree of adaptation, the scatter of fixation accounted for adaptation performance. This result raises the possibility that differences in adaptation in many studies could result from attention differences. It is thus also very important to control for attention in adaptation studies (see also gaze-contingent paradigms; e.g., Wilms et al. 2010). To our knowledge, controlling for attention has been employed in earlier studies on adaptation in autism by Ewing et al. (2013b) on face identity, Karaminis et al. (2015) on perceptual causality, Lawson et al. (2018) on eye-gaze direction and Rhodes et al. (2018) on facial expression. Our study on adaptation to the running speed of biological motion in autism is novel in combining the use of a secondary attention task with eye-tracking.

Our study is not without its shortcomings. We applied four exclusion criteria and thus excluded a considerable number of participants from our initial dataset to obtain a dataset that would allow measuring the adaptive coding of biological motion. The dual-task paradigm was also demanding, especially for younger participants. Finally, adaptation to biological motion in participants who were not able to attend to stimuli was also not explored in this study.

## Conclusion

Sensory differences have been included in the latest diagnostic criteria for autism (DSM-5; APA 2013) and represent some of the most puzzling features of the condition. The renewed interest in autistic sensory differences by researchers is prompted largely by the possibility that these and other non-social features of autism might be caused by fundamental differences in sensation and perception. Our results provide evidence that diminished adaptation, proposed to be one such fundamental difference, is not pervasive in autistic perception. Our findings demonstrate that more nuanced accounts of adaptation in autism are warranted, which address the potentially uneven adaptation profile in autism and its developmental implications (cf. Karaminis et al. 2015). The interplay between adaptation and attention is also important for a fuller understanding of autistic perception.

## Electronic supplementary material

Below is the link to the electronic supplementary material.

Supplementary material 1 (PDF 255 kb)

## Footnotes

1 We use ‘identify-first’ language (‘autistic person’) rather than person-first language (‘person with autism’), because it is the preferred term of autistic activists (e.g., Sinclair 1999) and many autistic people and their families (Kenny et al. 2016) and is less associated with stigma (Gernsbacher 2017).
