# Supplementary material for: Adaptation to the Speed of Biological Motion in Autism
Source: J Autism Dev Disord. 2019 Oct 19;50(2):373–85. doi: 10.1007/s10803-019-04241-4 (PMC6994433; doi:10.1007/s10803-019-04241-4)
Supplement: Supplementary file 1 — Supplementary material 1 (PDF 255 kb) [file 10803_2019_4241_MOESM1_ESM.pdf]

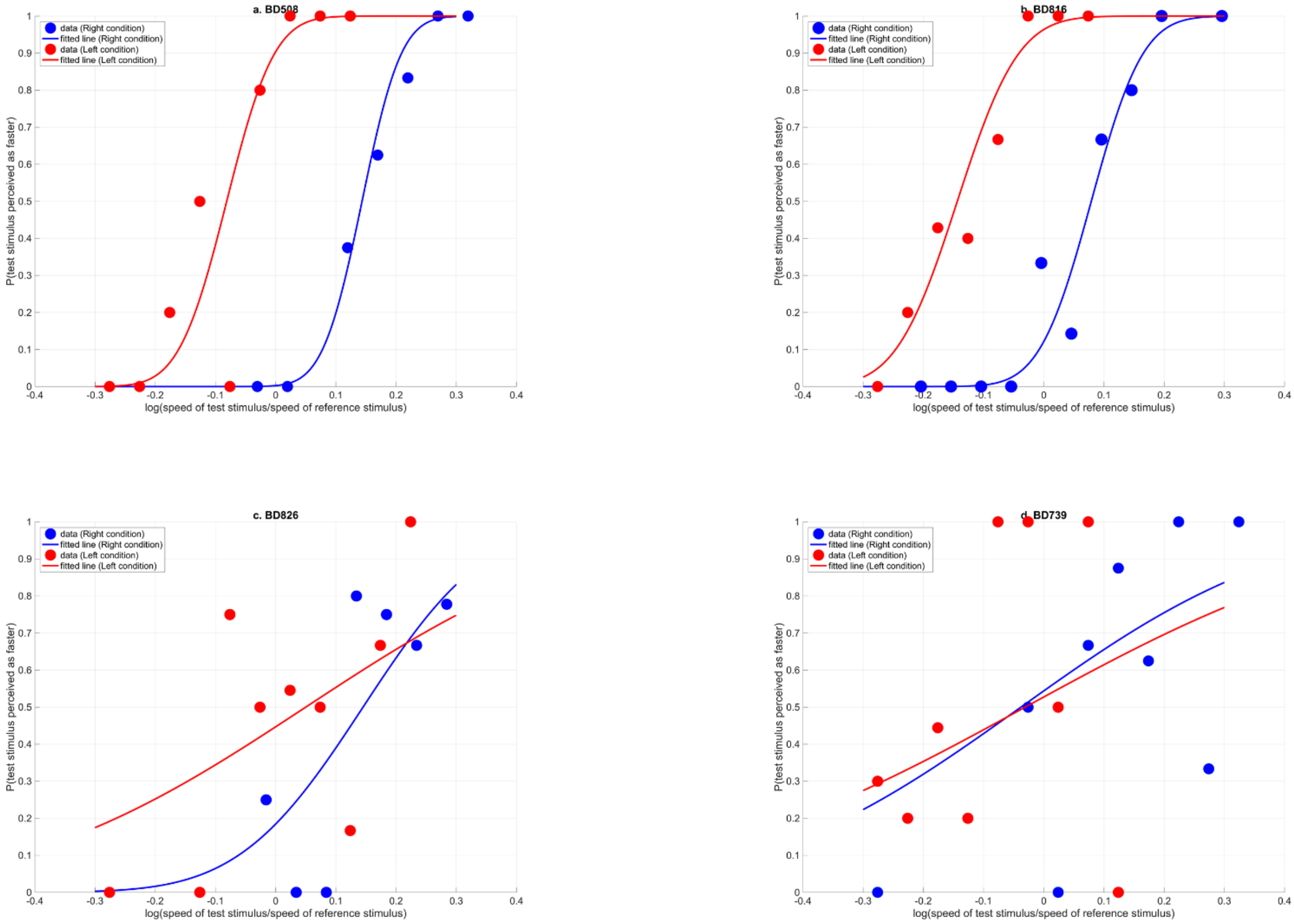

Supplementary Figure

Two examples of good (upper panels) and two examples of bad fits (lower panels) of cumulative Gaussian functions to data from the speed-discrimination task.
